# Photoperiodic regulation of dopamine signaling regulates seasonal changes in retinal photosensitivity in mice

**DOI:** 10.1038/s41598-021-81540-w

**Published:** 2021-01-19

**Authors:** Kousuke Okimura, Yusuke Nakane, Taeko Nishiwaki-Ohkawa, Takashi Yoshimura

**Affiliations:** 1 Institute of Transformative Bio-Molecules (WPI-ITbM), Nagoya University, Furo-cho, Chikusa-ku, Nagoya, 464-8601 Japan; 2 Laboratory of Animal Integrative Physiology, Graduate School of Bioagricultural Sciences, Nagoya University, Nagoya, 464-8601 Japan

**Keywords:** Circadian regulation, Sensory processing

## Abstract

At high latitudes, approximately 10% of people suffer from depression during the winter season, a phenomenon known as seasonal affective disorder (SAD). Shortened photoperiod and/or light intensity during winter season are risk factors for SAD, and bright light therapy is an effective treatment. Interestingly, reduced retinal photosensitivity along with the mood is observed in SAD patients in winter. However, the molecular basis underlying seasonal changes in retinal photosensitivity remains unclear, and pharmacological intervention is required. Here we show photoperiodic regulation of dopamine signaling and improvement of short day–attenuated photosensitivity by its pharmacological intervention in mice. Electroretinograms revealed dynamic seasonal changes in retinal photosensitivity. Transcriptome analysis identified short day-mediated suppression of the *Th* gene, which encodes tyrosine hydroxylase, a rate-limiting enzyme for dopamine biosynthesis. Furthermore, pharmacological intervention in dopamine signaling through activation of the cAMP signaling pathway rescued short day–attenuated photosensitivity, whereas dopamine receptor antagonists decreased photosensitivity under long-day conditions. Our results reveal molecular basis of seasonal changes in retinal photosensitivity in mammals. In addition, our findings provide important insights into the pathogenesis of SAD and offer potential therapeutic interventions.

## Introduction

At high latitudes with harsh winters, as in northern regions of the United States and Nordic countries, approximately 10% of the population suffer from depression in winter, also known as seasonal affective disorder (SAD)^1,2^. Typical symptoms of SAD include low mood, lethargy, sleep problems, disrupted circadian rhythms, social withdrawal, and changes in appetite and body weight. Because SAD leads to increased suicide rate in these northern regions, it represents a significant public health issue.

Shortening photoperiod and/or decreasing bright light exposure in winter are risk factors for SAD, and bright light therapy is used to treat the condition^1,3–5^. SAD patients exhibit reduced retinal photosensitivity in winter, as determined by electroretinogram (ERG) recordings, and recovers in summer or after treatment with bright light therapy^5–7^. Accordingly, the retina is thought to play a critical role in the pathogenesis of SAD. However, the underlying molecular basis of seasonal changes in retinal photosensitivity remains unclear. Although bright light therapy is an effective treatment, the most common side effects, such as eyestrain, headache, irritability, fatigue, nausea, and agitation, prevent patients from sitting in front of bright light apparatus^3,8^. Therefore, pharmacological intervention for reduced retinal sensitivity is required.

In this study, we sought to clarify the mechanisms underlying seasonal changes in photosensitivity, using mice as a model. Melatonin, whose secretion profile is controlled by ambient photoperiod, plays critical roles in the regulation of seasonality in mammals^9^. However, most inbred mice, including C57BL, DBA, and BALB/c, are insensitive to seasonal changes due to genetic defects in the melatonin biosynthesis pathway^10,11^. Therefore, we used melatonin-proficient, photoperiod-sensitive CBA/N mice in this study^12–14^. We recently demonstrated dynamic seasonal plasticity in photosensitivity and color perception in poikilothermal medaka fish and showed that these changes were temperature dependent^15^. Therefore, we examined the effects of changing photoperiod and temperature in this study. We first demonstrated that mice indeed exhibit reduced photosensitivity under winter-like conditions (short day and cool temperature) relative to summer-like conditions (long day and warm temperature), as revealed by ERG recordings. Time-series transcriptome analysis of the eye identified reduced expression of *Tyrosine hydroxylase* (*Th*) gene under winter-like conditions. Interestingly, retinal photosensitivity and *Th* expression were both photoperiod-dependent but temperature-independent. TH is a rate-limiting enzyme for dopamine biosynthesis, and retinal dopamine regulates retinal photosensitivity^16,17^. Finally, pharmacological intervention in dopamine signaling through activation of the cAMP signaling pathway rescued short day-attenuated photosensitivity, whereas dopamine receptor antagonists decreased photosensitivity under long-day conditions. These findings suggest possible therapeutic interventions for winter depression.

## Results

### Attenuated mesopic ERG under winter-like conditions

**Figure 1:**
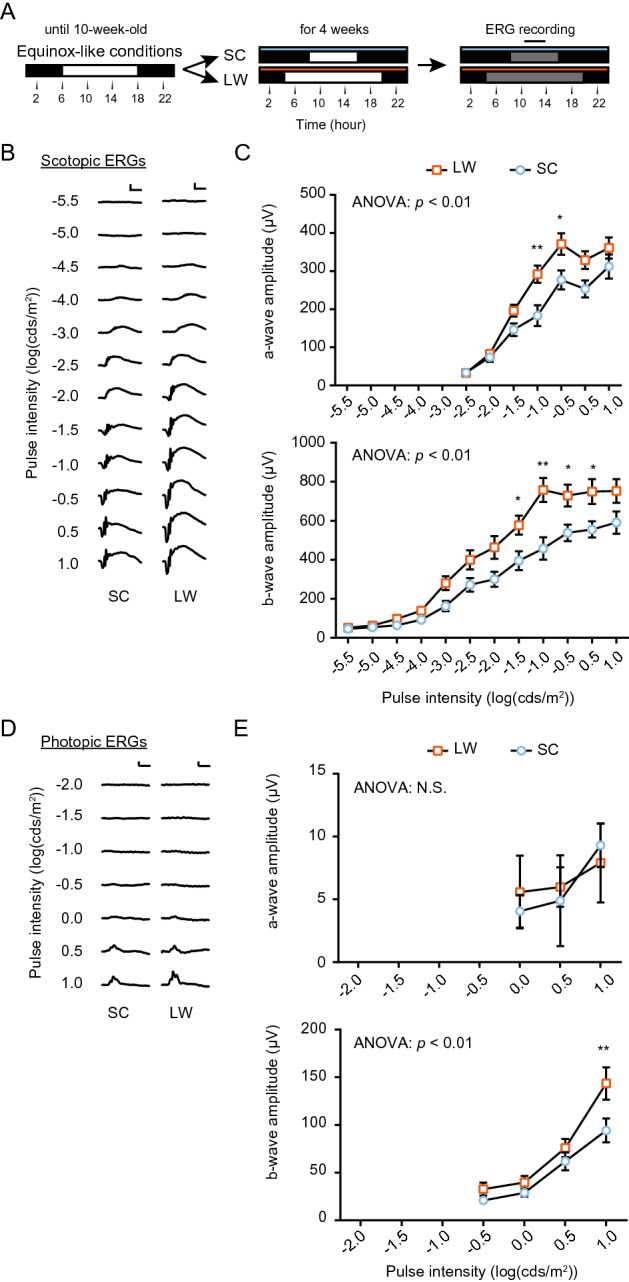
Seasonal changes in the mesopic ERG response in mice. (**A**) Flow of the experiment for ERG recording. Black and white bars indicate dark and light periods, respectively. Blue and orange bars indicate cool (10 °C) and warm (27 °C) conditions, respectively. SC: short day and cool temperature (winter-like condition); LW: long day and warm temperature (summer-like condition). ERG recording was conducted at around midday (black solid line) under darkness (gray bar) (i.e., dark adaptation). (**B**) Scotopic pulse ERGs elicited using 12 different intensities (from − 5.5 to 1.0 (log cds/m^2^)) in SC and LW mice (scale bar: 200 µV [vertical], 50 ms [horizontal]). (**C**) Intensity–response curves of mean scotopic a- and b-wave amplitudes. (Two-way ANOVA, effect of seasonal cues, *F*_(1, 154)_ = 23.73, *p* < 0.01 [a-wave]; *F*_(1, 264)_ = 57.19, *p* < 0.01 [b-wave], Bonferroni post hoc test: *p < 0.05, **p < 0.01; effect of pulse intensity, *F*_(6, 154)_ = 64.55, *p* < 0.01 [a-wave], *F*_(11, 264)_ = 72.29, *p* < 0.01 [b-wave]; effect of interaction, *F*_(6, 154)_ = 1.901, *p* = 0.0840 [a-wave], *F*_(11, 264)_ = 2.326, *p* < 0.01 [b-wave], means ± SEM, n = 12 [animal]). (**D**) Photopic pulse ERGs elicited using different intensities (from − 2.0 to 1.0 log (cds/m^2^)) in SC and LW mice (scale bar: 50 µV [vertical], 50 ms [horizontal]). (**E**) Intensity–response curves of mean photopic a- and b-wave amplitudes (two-way ANOVA, effect of seasonal cues, *F*_(1, 66)_ = 0.03748, *p* = 0.8471 [a-wave]; *F*_(1, 88)_ = 10.06, *p* = 0.0021 [b-wave], Bonferroni post hoc test: **p < 0.01; effect of pulse intensity, *F*_(2, 66)_ = 1.284, *p* = 0.2838 [a-wave], *F*_(3,88)_ = 38.56, *p* < 0.01 [b-wave]; effect of interaction, *F*_(2,66)_ = 0.1947, *p* = 0.8236 [a-wave], *F*_(3,88)_ = 1.877, *p* = 0.1394 [b-wave], mean ± SEM, n = 12 [animal]).

In order to investigate seasonal changes in photosensitivity in mammals, 10-week-old male CBA/N mice kept under 12-h light/12-h dark (12L12D) and 24 °C (equinox-like conditions) were transferred to long day (LD) and warm, summer-like conditions (LW: 16-h light/8-h dark, 27 °C) or short day (SD) and cool, winter-like conditions (SC: 8-h light/16-h dark, 10 °C) for 4 weeks. We used only male mice in the present study because the estrus cycle is known to influence the retinal physiology, including the ERG^18^. At 14 weeks of age, mice were dark-adapted overnight, and scotopic (dark-adapted) and photopic (light-adapted) ERGs were recorded at the middle of the light phase of each photoperiod (Fig. 1A)^19^. ERGs consist mainly of two waves; when the eyes are exposed to a light flash, a negative deflection a-wave initially appears, reflecting photoreceptor activation; subsequently, a positive deflection b-wave arises, reflecting the activity of interneurons, including bipolar cells and Müller cells. SC mice exhibited a significant decrease in scotopic ERGs than LW mice in both a-wave amplitudes (two-way ANOVA, *p* < 0.01, n = 12 [animal]) and b-wave amplitudes (two-way ANOVA, *p* < 0.01, n = 12 [animal]) when light pulse intensities were higher than − 1.0 log(cds/m^2^) and − 1.5 log(cds/m^2^), respectively (Fig. 1B, C). When a pulse intensity of − 1.0 log(cds/m^2^) was given, the most significant differences in the average amplitude were recorded in both a- and b-waves. At this pulse intensity, a- and b-wave amplitudes in SC mice were 37% and 40% lower than in LW mice, respectively. In mice, irradiances higher than − 1.28 log(cds/m^2^) under scotopic conditions induce not only rod responses but also cone responses, known as mesopic responses^20,21^. When we examined photopic ERGs, we observed no significant change in the a-wave, whereas a significant change was observed at the highest intensity of 1.0 log(cds/m^2^) (two-way ANOVA, *p* < 0.01, n = 12; Fig. 1D, E). Based on these observations, winter-like SC conditions induced attenuated ERGs at mesopic light levels.

### Identification of seasonally regulated genes in the mouse eye

**Figure 2:**
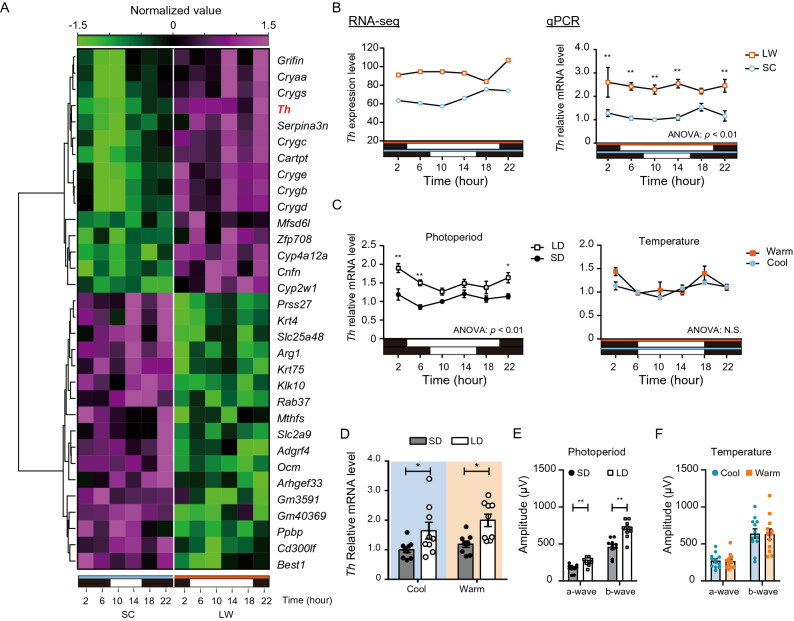
Photoperiodic regulation of *Th* expression and mesopic ERG. (**A)** Hierarchical clustering of 15 genes upregulated under LW conditions and 17 genes upregulated under SC conditions. Heatmap was generated in R (version 4.0.3) with the heatmap.2 function from gplots package (https://biocorecrg.github.io/CRG_RIntroduction/heatmap-2-function-from-gplots-package.html). (**B**) Differential expression of *Th* gene detected by RNA-seq (n = 2) (left) and qPCR (n = 3) (right) in the eyes of the mice kept under SC and LW conditions. Two-way ANOVA and Bonferroni post hoc test were used for qPCR analysis (two-way ANOVA, effect of seasonal cues, *F*_(1, 24)_ = 77.91, *p* < 0.01, Bonferroni post hoc test: **p < 0.01; effect of time, *F*_(5,24)_ = 0.3711, *p* = 0.8634; effect of interaction, *F*_(5, 24)_ = 0.6346, *p* = 0.6753, mean ± SEM, n = 3). (**C**) Effects of photoperiod (left) or temperature (right) on *Th* gene expression measured by qPCR (two-way ANOVA, effect of photoperiod, *F*_(1, 24)_ = 53.76, *p* < 0.01; effect of temperature, *F*_(1, 24)_ = 3.647, Bonferroni post hoc test: *p < 0.05, **p < 0.01; effect of time, *F*_(5,24)_ = 4.129, *p* < 0.01 [photoperiod], *F*_(5,24)_ = 6.008, *p* < 0.01 [temperature]; effect of interaction, *F*_(5,24)_ = 1.670, *p* = 0.1801 [photoperiod], *F*_(5,24)_ = 1.600, *p* = 0.1982 [temperature], mean ± SEM, n = 3). (**D**) Effects of photoperiod and temperature on *Th* expression (two-way ANOVA, effect of photoperiod, *F*_(1, 32)_ = 14.44, *p* = 0.0006, Bonferroni post hoc test: *p < 0.05; effect of temperature, *F*_(1,32)_ = 1.987, *p* = 0.1683; effect of interaction, *F*_(1,32)_ = 0.1900, *p* = 0.6658, mean ± SEM, n = 9). (**E**) Effect of photoperiod on mesopic responses (Student’s *t*-test, *t*_(18)_ = 3.825 [a-wave], *t*_(18)_ = 4.732 [b-wave], **p < 0.01, mean ± SEM, n = 10). (**F**) Effect of temperature on mesopic responses (Student’s *t*-test, *t*_(22)_ = 0.1534 [a-wave], *t*_(22)_ = 0.08799 [b-wave], *p* > 0.05, mean ± SEM, n = 10–12). Mesopic ERG was measured using a − 1.0 log(cds/m^2^) light pulse.

To reveal the mechanisms underlying seasonal changes in ERG responses, we next examined RNA-seq analysis using time series samples of the whole eye (collected every 4 h, 6 time points, n = 2) under SC and LW conditions. If the minimum expression level of a gene among all the time points in one condition (e.g., SC or LW) was higher than the maximum expression level in another condition, the gene was defined as differentially expressed gene (DEG). This analysis identified 15 LW-upregulated and 17 SC-upregulated DEGs, respectively (Fig. 2A). Unlike medaka fish^15^, we did not detect clear differences in rhodopsin family genes (Supplementary Fig. S1). However, we observed downregulation of *Th* gene under winter-like SC conditions (Fig. 2A, B). The differential expression of *Th* was verified by real-time quantitative PCR (qPCR) (two-way ANOVA; *p* < 0.01, n = 3; Fig. 2B). TH is a rate-limiting enzyme for dopamine biosynthesis. It is well established that diurnal dopamine secretion from dopaminergic amacrine cells plays key roles in retinal photosensitivity^16,17^.

We next sought to determine which seasonal cue (e.g., photoperiod or ambient temperature) regulates *Th* expression level by qPCR. Although ambient temperature had no significant effect (two-way ANOVA, *p* = 0.0682, n = 3), *Th* was differentially expressed under different photoperiodic conditions (two-way ANOVA, *p* < 0.01, n = 3) (Fig. 2C). We further confirmed this result by exposing mice to different conditions: short day and cool (SC), short day and warm (SW), long day and cool (LC), and long day and warm (LW). When eyes were collected at the middle of the light phase, *Th* expression was higher under long-day (LC and LW) conditions than short-day (SC and SW) conditions irrespective of the temperature, indicating that photoperiod is the primarily seasonal cue regulating *Th* expression (two-way ANOVA, *p* = 0.0006, n = 9; Fig. 2D).

To further distinguish the effect of photoperiod and temperature on mesopic ERGs, mice were kept under SD or LD conditions at 24 °C for 4 weeks, and mesopic ERGs were measured using a light pulse of − 1.0 log(cds/m^2^) (Fig. 2E). SD mice exhibited lower mesopic ERG responses in both a-wave and b-wave amplitudes relative to LD mice (Student’s *t*-test, *p* < 0.01, n = 12). Average amplitudes of a- and b-waves in mesopic responses of SD mice were 35% lower than those of LD mice. By contrast, no significant change was detected in mice kept under equinox photoperiod (12L12D) under cool and warm conditions (Student’s *t*-test, *p* > 0.05, n = 12; Fig. 2F). Taken together, both *Th* expression level and mesopic ERG responses were regulated by changes in photoperiod, regardless of ambient temperature.

To test the involvement of melatonin, we also examined the effects of SD and LD in melatonin-deficient C57BL/6J mice, and photoperiodic regulation of both *Th* and b-wave amplitude was observed at the middle of the light phase in each photoperiodic condition (Supplementary Fig. S2).

### Pharmacological intervention in seasonally regulated retinal photosensitivity

**Figure 3:**
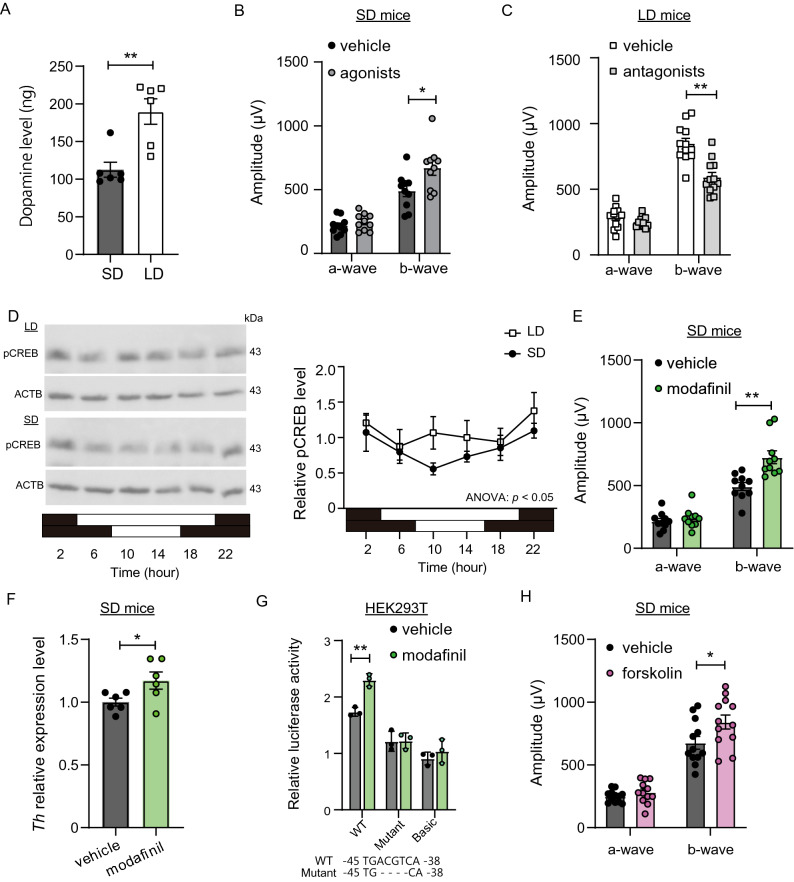
Pharmacological intervention of dopamine signaling regulates mesopic b-wave amplitude. (**A**) Increase in dopamine in the eyes of mice by long-day conditions measured by ELISA. (Student’s *t*-test, *t*_(10)_ = 3.943, **p = 0.0028, mean ± SEM, n = 6). (**B**) Effect of dopamine receptor (D1/D4) agonist cocktail on mesopic ERG responses in SD mice (Student’s *t*-test, *t*_(18)_ = 0.9807 [a-wave], t_18_ = 2.520 [b-wave], *p = 0.0214 mean ± SEM, n = 10). (**C**) Effect of dopamine receptor (D1/D4) antagonist cocktail on mesopic ERG responses in LD mice (Student’s *t*-test, *t*_(22)_ = 1.386 [a-wave], *t*_(22)_ = 4.676 [b-wave], **p < 0.01, mean ± SEM, n = 12). (**D**) Western blotting for pCREB and ACTB in eyes of SD and LD mice (left). Relative pCREB value was normalized against ACTB level (right) (two-way ANOVA, effect of photoperiod, *F*_(1, 24)_ = 4.353, *p* = 0.0477; effect of time, *F*_(5,24)_ = 1.803, *p* = 0.1503; effect of interaction, *F*_(5,24)_ = 0.8554, *p* = 0.8554, mean ± SEM, n = 3). To quantify band intensity, three different gels were used (see Supplementary Fig. 3). (**E**) Effect of modafinil on mesopic ERG responses in SD mice (Student’s *t*-test, *t*_(18)_ = 0.7161 [a-wave], *t*_(18)_ = 3.855 [b-wave], **p = 0.0012, mean ± SEM, n = 10). (**F**) Effect of modafinil on *Th* expression level measured by qPCR (Student’s *t*-test, *t*_(10)_ = 2.266, *p = 0.0469, mean ± SEM, n = 6). (**G**) Promoter activity of mouse *Th* was measured using HEK293T cells. Wild-type and mutant reporters fused to the *luciferase* gene were assayed for their activities in response to modafinil. CRE sequences of wild type (WT) and mutant (Mutant) are shown at bottom. Basic: pGL4.19-basic empty vector. (Student’s *t*-test, *t*_(4)_ = 7.199 [WT], *t*_(4)_ = 0.06143 [Mutant], *t*_(4)_ = 0.9412 [Basic], **p = 0.0020, mean ± SEM, n = 3) (**H**) Effect of eyedrop topical administration of forskolin on mesopic ERG responses in SD mice (Student’s *t*-test, *t*_(22)_ = 1.145 [a-wave], *t*_(22)_ = 2.181 [b-wave], *p = 0.0402, mean ± SEM, n = 12). Mesopic ERG was measured using a − 1.0 log(cds/m^2^) light pulse.

Reduced *Th* expression level appeared to affect ocular dopamine level under SD conditions. Indeed, SD mice had a lower ocular dopamine level than LD mice (Student’s *t*-test, *p* = 0.0028, n = 4; Fig. 3A). The mammalian retina contains four types of dopamine receptors^17,22^. Among them, dopamine D1 receptors expressed in cone ON-bipolar cells and horizontal cells, and D4 receptors expressed in photoreceptors play important roles in retinal photosensitivity^16,17^. Therefore, we next assessed the effect of pharmacological intervention in dopamine signaling. Intraperitoneal (i.p.) injection of a cocktail of agonists for D1 and D4 dopamine receptors (SKF38393 and PD168077, 1 mg/kg each) 1 h before the ERG recording significantly improved the b-wave amplitude in SD mice (Student’s *t*-test, *p* = 0.0214, n = 10; Fig. 3B). On the other hand, i.p. injection of a cocktail of antagonists for D1 and D4 receptors (SCH23390 and L-745870, 1 mg/kg each) 1 h before the ERG recording decreased b-wave amplitude in LD mice (Student’s *t*-test, *p* < 0.01, n = 12; Fig. 3C).

The cAMP-responsive element (CRE) (TCACGTCA) in the promoter region of *Th* is responsible for promoting basic and induced *Th* gene expression^23^, and phosphorylated CREB (pCREB) binds CRE to induce transcription. Western blotting revealed that the pCREB level in the eye was different between LD mice and SD mice (two-way ANOVA, *p* = 0.0477, n = 3; Fig. 3D, Supplementary Fig. S3). Modafinil inhibits dopamine reuptake^24^ and engages the activation of pCREB^25^. Gavage administration of modafinil (64 mg/kg, 3 times per day for 3 days; Supplementary Fig. S4) improved b-wave amplitude (Student’s *t*-test, *p* = 0.0012, n = 10; Fig. 3E) and increased *Th* gene expression in the eyes of SD mice in vivo (Student’s *t*-test, *p* = 0.0469, n = 6; Fig. 3F). To further investigate whether the CRE sequence is indeed involved in the regulation of *Th* gene by modafinil, we analyzed the activity of the *Th* promoter transfected into the HEK293T cell line. Modafinil administration induced expression of *Th* reporter activity in the wild-type (WT) construct relative to the vehicle control (Student’s *t*-test, *p* = 0.0020, n = 3). However, when the CRE sequence was mutated, induction by modafinil was not observed (Student’s *t*-test, *p* = 0.9540, n = 3; Fig. 3G). Induction of reporter activity was also not observed in the ‘basic’ empty construct (Student’s *t*-test, *p* = 0.3999, n = 3). These results suggest that induction of *Th* by modafinil involves a cAMP signaling pathway.

Forskolin is well known to increase the cAMP level and hence CREB activation. Eye-drop topical administration of forskolin in SD mice (1% w/v, 3 times per day for 3 days; Supplementary Fig. S4) rescued short day–attenuated mesopic b-wave amplitude (Student’s *t*-test, *p* = 0.0402, n = 12; Fig. 3H). Together, these results suggest that pharmacological intervention in the dopamine signaling pathway can rescue seasonally regulated retinal photosensitivity in mice.

## Discussion

Reduced light exposure during the winter season is considered to be a risk factor for SAD^1,4,5^. Although SAD patients exhibit reduced photosensitivity during depressive episodes^5–7^, the molecular basis of the condition remains unclear. In this study, we demonstrated seasonal changes in retinal photosensitivity in mice, as determined by measurement of ERGs (Fig. 1), indicating that mice could be an excellent model to uncover its molecular mechanism. Although organisms are exposed to significant changes under both ambient photoperiod and temperature at high latitudes, we found that changes in photoperiod, but not temperature, affected the retinal photosensitivity in mice (Fig. 2). In a previous study, we observed seasonal changes in retinal photosensitivity in medaka fish^15^. In this species, however, retinal photosensitivity was regulated by ambient temperature but not by photoperiod. It is reasonable to speculate that these differences are related to the differences between homeothermic and poikilothermic animals. In addition to the changes in photosensitivity, our previous study demonstrated seasonal changes in color perception in medaka fish. Functional analyses revealed that the seasonal changes in color perception were caused by dynamic seasonal changes in the expression of rhodopsin family genes^15^. Interestingly, seasonal changes in color perception have also been reported in healthy humans^26^. Therefore, we hypothesized that expression of rhodopsin family genes is also affected by seasonal cues in mammals. In contrast to our prediction, however, our RNA-seq analysis of SC and LW mice failed to detect clear expression changes in the rhodopsin family genes in mice (Supplementary Fig. S1). Medaka fish are seasonal breeders that nuptial coloration during breeding season to attract mates. However, mice are non-seasonal breeders and do not change body coloration throughout the seasons. Therefore, it would be interesting to examine expression changes in rhodopsin family genes in seasonally breeding mammals that exhibit color ornamentation during the mating season, such as the red skin coloration in macaques^27^, in future studies.

Although we did not observe changes in the expression of rhodopsin family genes, we detected clear seasonal changes in *Th* expression and dopamine level in the eye (Figs. 2 and 3). Furthermore, pharmacological activation and inactivation of dopamine signaling regulated photoperiodically controlled retinal sensitivity (Fig. 3). These results are consistent with the notion that the retinal dopamine signaling plays a critical role in the regulation of retinal photosensitivity^17^. Abnormal melatonin secretion is observed in SAD patients during depression episode^28^. Furthermore, nocturnal melatonin suppresses retinal dopamine release^29^, and dopamine suppresses melatonin production in the retina^30,31^. Therefore, mutual inhibitory regulation of retinal melatonin and dopamine is speculated to be involved in the pathogenesis of SAD (the ‘retinal melatonin dopamine hypothesis’)^32^. In this study, we observed photoperiodic regulation of both *Th* and retinal photosensitivity in melatonin-deficient C57BL mice (Supplementary Fig. S2), suggesting that melatonin is not necessary for seasonal regulation of retinal photosensitivity.

A previous study reported that photoperiod during the perinatal stage has an enduring impact on retinal dopaminergic systems and visual functions in mice^16^. This study further demonstrated that the changes in photoperiod during adulthood also affect ocular dopamine signaling, suggesting that photoperiodic regulation of retinal photosensitivity is more plastic than previously considered. This finding fits well with previous reports that the reduced photosensitivity observed in SAD patients during winter recovers in summer or after bright light therapy^5–7^.

Although the selective serotonin reuptake inhibitor fluoxetine has been used for the treatment of SAD, this practice may not be supported by sufficient evidence^33^. We recently reported that the traditional Chinese medicine celastrol can rescue the winter depression-like behavior in medaka fish through activation of NRF2 antioxidant pathway^34^. Therefore, drugs targeting the NRF2 antioxidant signaling pathway could be potential therapeutic targets for SAD in the future. Often, however, treatment of multifactorial psychiatric disorders with just one drug or monotherapy is insufficient, and comedication therapy is more effective. As mentioned above, although bright light therapy is effective for the treatment of SAD patients, side effects prevent patients from sitting in front of bright light apparatus^3,8^; therefore, pharmacological interventions that take the place of bright light therapy are expected. In this study, we demonstrated that pharmacological activation of retinal dopamine signaling rescues photoperiodically regulated retinal photosensitivity in mice through a cAMP signaling pathway. Forskolin is a well-known bioactive diterpene derivative that increases cAMP level and hence CREB activation. Oral administration of food supplements containing forskolin restore ERGs of open glaucoma patients^35^. Furthermore, intraocular irrigation of forskolin increases ERG amplitude in rabbits^36^. As in the case of the blood–brain barrier, the delivery of compounds into the retina remains a challenge because of the blood–retinal barrier^37,38^. However, since forskolin penetrates the blood–brain barrier^39,40^, we examined the effect of topical administration of forskolin in SD mice, and found that this treatment improved short day–attenuated mesopic b-wave amplitude (Fig. 3).

Growing evidence suggests that photo-stimuli received by the eye affect multiple brain functions, including circadian rhythms and mood^41,42^; hence, it has been suggested that the retina plays a significant role in the pathogenesis of SAD^5,32,43^. This idea is not surprising considering that the retina, like other regions of the central nervous system, originates from the neural tube. Indeed, the retina is a true part of the brain and thus a gateway to the brain^44^. Therefore, in addition to the development of antidepressants acting on the central nervous system, drugs targeting retinal dopaminergic signaling pathways could offer another therapeutic target for treating or preventing SAD.

## Materials and methods

### Animals

Male 10-week-old CBA/N and C57BL/6J mice were obtained from a local dealer (Nihon SLC, Hamamatsu). Mice were kept under following conditions using housing systems (LP-30CCFL-8CTAR, NK system; Nippon Medical & Chemical Instruments, Osaka, Japan) for 4 weeks: LW (long day and warm temperature; 16-h light/8-h dark and 27 °C), LC (long day and cool temperature; 16-h light/8-h dark and 10 °C), SC (short day and cool temperature; 8-h light/16-h dark and 10 °C), SW (short day and warm temperature; 8-h light/16-h dark and 27 °C), LD (long day; 16-h light/8-h dark and 24 °C), SD (8-h light/16-h dark and 24 °C), Cool (12-h light/12-h dark and 10 °C), or Warm (12-h light/12-h dark and 27 °C) conditions. In Figs. 1 and 2A,B, mice were divided into two groups and maintained under LW and SC. In Fig. 2C (left) and 2E, mice were divided into two groups and maintained under SW and LW. In Fig. 2C (right) and 2F, mice were divided into two groups and maintained under Cool and Warm conditions. In Fig. 2D, mice were divided into 4 groups and maintained under LW, LC, SW, and SC, respectively. Food and water were provided to the animals ad libitum. Animals were treated in accordance with the guidelines of Nagoya University, and all experimental procedures were approved by the Animal Experiment Committee of Nagoya University.

### ERG recordings

The procedures for electroretinograms (ERGs) were performed according to previously reported paper^45^. Mice were anesthetized with i.p. injection of ketamine and xylazine at midday under darkness (i.e., dark adaptation). Tropicamide (0.5%) and phenylephrine HCl (0.5%) were given with eye drops to dilate the pupils just before starting the ERG recording. ERGs were recorded under dim red light, and the mice were placed on a heating pad during ERG recordings. ERGs were recorded with a gold wire loop electrode (N1530NNC; Mayo Corporation, Inazawa, Japan) on the cornea with a gold wire reference electrode placed on the sclera. The ERGs were automatically averaged with a computer-assisted signal-averaging system (MLS060/8 PowerLab; ADInstruments, Colorado Springs, CO, USA). A Ganzfeld bowl with a xenon source (LS-100; Mayo Corporation) was used for pulse stimulation.

For the scotopic ERGs, two to six responses were averaged with an interval of 10 s for each pulse intensity. For the photopic ERGs, 20 to 30 responses were averaged with an interval of 1 s for each pulse intensity. Twelve equal pulse-intensity steps, ranging from − 5.5 to 1.0 log(cds/m^2^) were used to record scotopic ERGs. For photopic ERGs, seven equal pulse-intensity steps, ranging from − 2.0 to 1.0, were used with rod-desensitizing white adapting halogen background light of 39.8 cd/m^2^. For photopic ERGs, mice were light-adapted for 10 min before starting the first intensity step.

### Tissue collection

Eyes were collected at 6 time points (2, 6, 10, 14, 18, and 22 h after midnight), or at midday. Samples were immediately frozen in a 1.5-ml tube on liquid nitrogen and stored at − 80 °C until RNA extraction for gene expression analyses and protein extraction for western blot analysis.

### Gene expression analyses

Total RNA was purified with RNeasy lipid mini tissue kit (Qiagen, Hilden, Germany) with DNase I. Extracted RNA was stored at − 80 °C. For strand-specific RNA-seq analysis, in order to produce paired-end libraries, extracted total RNA was treated using a TruSeq Stranded mRNA LT Sample Prep kit (Illumina, San Diego, CA, USA) and sequenced at a read depth of ~ 6.42 Gb per sample with 100-bp reads on an Illumina HiSeq 4000 system (Illumina). Read quality control was checked with FastQC and filtered using the software SOAPnuke. Reads were mapped to the mouse genome using HISAT2. Gene expression levels were calculated by RNA-Seq by Expectation–Maximization (RSEM).

For real-time quantitative PCR (qPCR), reverse transcription was performed on total RNA (100 ng) using the ReverTra Ace qPCR RT Kit (Toyobo, Osaka, Japan). TaqMan Master mix (Applied Biosystems, Foster City, CA, USA), TaqMan probes of *Th* (Mm00447557_m1), or *Eif1ad* (Mm01233367_m1), and 2 µl of synthesized cDNA were mixed in a 20-µl volume. qPCR was performed on a QuantStudio 3 Real-Time PCR Systems (Applied Biosystems). Because expression levels of *Gapdh* and *Actb* were influenced by environmental cues in this study, the housekeeping gene *Eif1ad* (*eukaryotic translation initiation factor 1A domain containing*) was used as an internal control.

### ELISA to measure dopamine

Eyes were collected at midday from mice kept under SD or LD conditions for 4 weeks. Samples were immediately frozen in a 1.5-ml tube on liquid nitrogen and stored at − 80 °C until dopamine measurement. Frozen eyes were homogenized in 1 ml of 0.01 N HCl in the presence of EDTA and sodium metabisulfite. Dopamine levels were measured in homogenized samples (200 µL volume) using the Dopamine Research ELISA (ImmuSmol, Bordeaux, France).

### Drug administration

Dopamine receptor agonist cocktail contained D1 receptor agonist (SKF38393; Abcam, Cambridge, UK) and D4 receptor agonist (PD168077; Tocris, Bristol, UK) dissolved in saline. Mice kept under SD conditions were i.p. injected with dopamine agonist cocktail (1 mg/kg) 1 h before the ERG recording under dim red light, as described previously^18^. Dopamine receptor antagonist cocktail contained D1 receptor antagonist (SCH 23390; Tocris) and D4 receptor antagonist (L-745870; Abcam) (1 mg/kg each) dissolved in saline. Mice kept under LD conditions were i.p. injected with dopamine antagonist cocktail 1 h before ERG recording under dim red light. Based on previous reports^46,47^, modafinil (M6940; Sigma-Aldrich, St. Louis, MO, USA), dissolved in 0.1% DMSO/saline, was injected by gavage (64 mg/kg, 4 h apart, three times each day during daytime for 3 days). Forskolin (66575-29-9; Wako, Osaka) dissolved in saline (1%) was administered to the eyes (2 µl each) as eye-drops using a pipet (4 h apart, three times each day during daytime for 3 days). The effects of topical administration of forskolin were examined twice by different experimenters, and consistent results were obtained in both experiments.

### Western blot analysis

Frozen eyes were lysed with ice-cold radioimmunoprecipitation (RIPA) buffer containing protease and phosphatase inhibitors. Sonicated lysate was centrifuged (12,000 g, 4 °C, 15 min), and the supernatant was collected. Eye homogenates were mixed with 2-mercaptoethanol and resolved by SDS-PAGE and transferred to activated PVDF membranes. The PVDF membranes were blocked with 2.5% skim milk in Tris-buffered saline with Tween 20 (TBST) for 1 h at room temperature. Phospho-CREB primary antibody (1:1,000) (#9189; Cell Signaling Technology, Danvers, MA, USA), dissolved in 5% bovine serum albumin in TBST, was incubated 16 h at 4 °C. Membranes were washed with TBST. Secondary rabbit antibody (1:2000), dissolved in 2.5% skim milk in TBST, was incubated for 1 h at room temperature. Washed membranes with TBST was incubated with ECL prime (GE Healthcare, Chicago, IL, USA) for 15 min at room temperature and imaged on a LAS3000 (Fuji Film, Tokyo, Japan). ACTB primary antibody (1:1,000) (sc-47778; Santa Cruz Biotechnology, Dallas, TX, USA) was used as an internal control after pCREB signals were eliminated by incubation in H_2_O_2_. The sizes of the protein were determined by Amersham ECL Rainbow Marker-Full Range (GE Healthcare). Quantification of immunoblots was conducted with Image J (https://imagej.nih.gov/ij/index.html).

### Constructs, transfection, and luciferase assay

The 963-bp 5′-flanking region of mouse *Th* was subcloned into pGL4.19-basic vector (Promega, Madison, WI, USA) using 5′-TTTGGTACCGTTTCCTTGGCTGAGGAAGCT-3′ and 5′-AACAAAGCTTAGTGCAAGCTGGTGGTCCCG-3′. A deletion construct of CRE was generated by deletion of the central four nucleotides (−45: TGACGTCA—> TGCA), as previously described^48,49^ using the KOD mutagenesis kit (Toyobo). Constructs were confirmed by direct sequencing (ABI 3100; Applied Biosystems).

The HEK293T cells were plated in 96-well plates at a density of ~ 2 × 10^6^ cells per well in 125 µl DMEM. *Th* promoter-luciferase construct (133 ng) was co-transfected with *Renilla* luciferase (19 ng) using Xfect reagent (Takara Bio, Kusatsu, Japan). After 24 h, cells were washed with DMEM, and 75 µl of DMEM was added to each well. Modafinil (0.1 mM, dissolved in 0.1% DMSO/DMEM) was added into wells 12 h after washing^50^. Sixteen hours later, luciferase activities were measured using the Dual-Glo luciferase assay (E2920; Promega) with SoftMax Pro (Molecular Devices, San Jose, CA, USA). Firefly relative luciferase activity was normalized against *Renilla* activity.

### Statistical analysis

Data are presented as means ± SEM generated by GraphPad Prism 8 (https://www.graphpad.com/scientific-software/prism/). Significance of differences between groups was evaluated by Student’s *t*-test. In the case of three or more groups, two-way ANOVA and post hoc test was conducted in GraphPad Prism 8.

## Supplementary Information

Supplementary Information 1.

## Data Availability

The RNA-seq data are available at NCBI Gene Expression Omnibus [accession number GSE156141].
