# Supplementary material for: Photoperiodic regulation of dopamine signaling regulates seasonal changes in retinal photosensitivity in mice
Source: Sci Rep. 2021 Jan 19;11:1843. doi: 10.1038/s41598-021-81540-w (PMC7815869; doi:10.1038/s41598-021-81540-w)
Supplement: Supplementary file 1 — Supplementary Information 1. [file 41598_2021_81540_MOESM1_ESM.pdf]

Supplementary Information

Photoperiodic regulation of dopamine signaling regulates seasonal changes in retinal photosensitivity in mice

Kousuke Okimura[1,2], Yusuke Nakane[1,2], Taeko Nishiwaki-Ohkawa[1,2], Takashi Yoshimura[1,2]

[1]Institute of Transformative Bio-Molecules (ITbM), Nagoya University, Nagoya 464-8601, Japan

[2]Laboratory of Animal Integrative Physiology, Graduate School of Bioagricultural Sciences, Nagoya University, Nagoya 464-8601, Japan

[*]Correspondence:
Takashi Yoshimura, Ph.D., FRSB
Professor of Integrative Physiology
Institute of Transformative Bio-Molecules (WPI-ITbM)
Nagoya University
Furo-cho, Chikusa-ku, Nagoya 464-8601, Japan
E-mail: takashiy@agr.nagoya-u.ac.jp

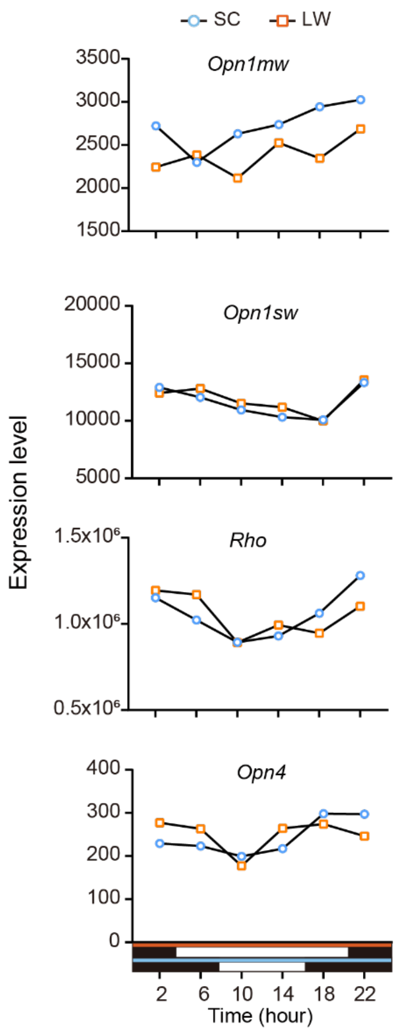

Supplementary Figure 1. Temporal expression profiles of rhodopsin family genes in SC and LW mouse eyes. Results of RNA-seq analysis for expression of *Opsin 1 middle-wave* (*Opn1mw*), *Opsin 1 short-wave* (*Opn1sw*), *Rhodopsin* (*Rho*), and *Melanopsin* (*Opn4*). (mean, n = 2).

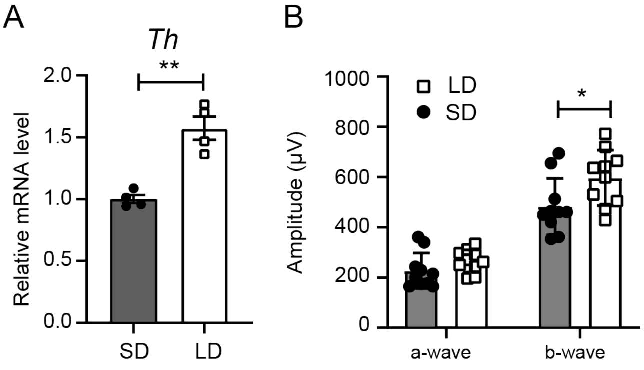

Supplementary Figure 2. Photoperiodic regulation of *Th* expression and ERG response in melatonin-deficient mice. Effects of short day (SD) and long day (LD) on ocular *Th* expression. (**A**) (Student's *t*-test, $t_{(6)} = 5.717$, $^{**}p = 0.0012$, mean ± SEM, n = 4) and mesopic ERG response. (**B**) (Student's *t*-test, $t_{(18)} = 1.466$ [a-wave], $t_{(18)} = 2.280$ [b-wave], $^{*}p = 0.0350$, mean ± SEM, n = 10) in male C57BL/6J mice. Mesopic ERG was measured using a −1.0 log(cds/m^2) light pulse.

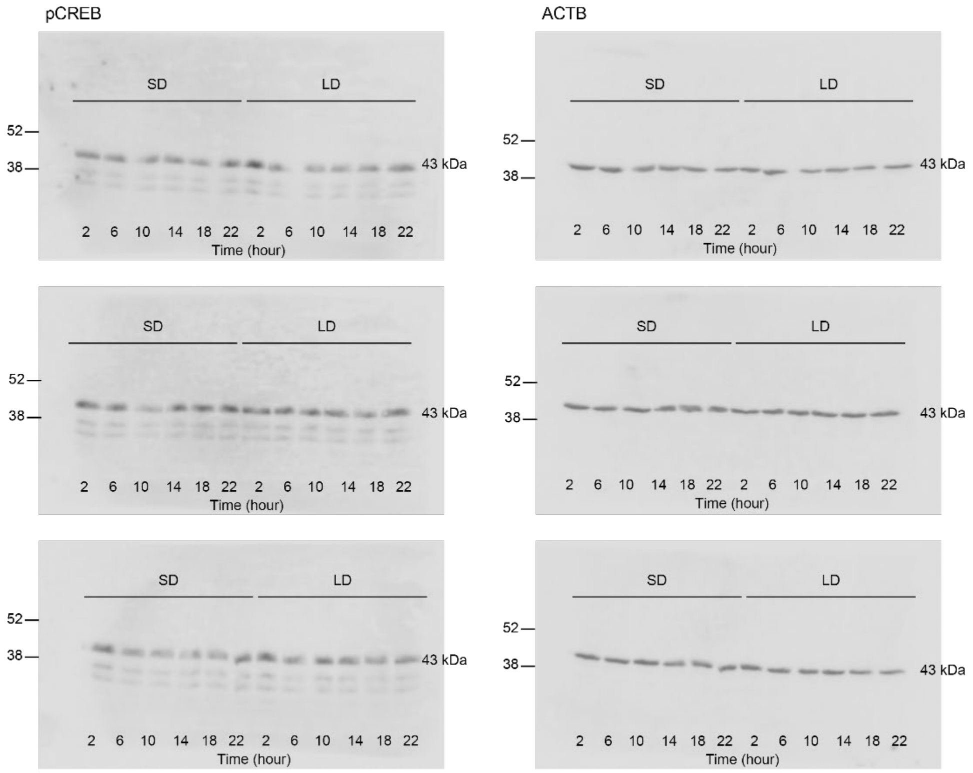

Supplementary Figure 3. Western blot analysis of pCREB in eyes of SD and LD mice. Three independent experiments were performed.

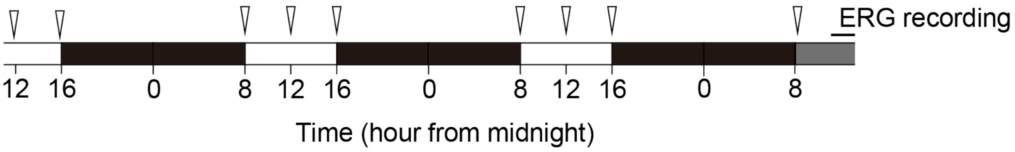

Supplementary Figure 4. Timelines of modafinil and forskolin administrations. The black and white bars indicate dark and light periods, respectively. ERG recordings were conducted at approximately midday (black solid line) above darkness (gray bar) (i.e., dark adaptation). The arrow head indicates the time points of drug administration.
